# Challenges in managing upper gastrointestinal bleeding secondary to primary squamous cell carcinoma of the pancreas: a case report and literature review

**DOI:** 10.1186/s40792-023-01663-2

**Published:** 2023-05-12

**Authors:** Colin Chan-Min Choi, Yasser Arafat, Maryam Shamassi, Julian Choi

**Affiliations:** 1 Department of Upper Gastrointestinal/Hepatobiliary Surgery, Western Health, Footscray, VIC Australia; 2 Department of Surgery, Western Precinct, University of Melbourne, Melbourne, Australia; 3 Department of Colorectal and General Surgery, Western Health, Footscray, VIC Australia; 4 Department of Anatomical Pathology, Western Health (Dorevitch Pathology), Footscray, VIC Australia

**Keywords:** Pancreatic squamous cell carcinoma, Gastrointestinal bleeding, New-onset diabetes mellitus

## Abstract

**Background:**

Primary pancreatic squamous cell carcinoma (SCC) is a rare type of pancreatic cancer, with an incidence of 5% of all pancreatic cancers. This condition is associated with a poor prognosis, and no optimal treatment has been established (Zhang et al. in Medicine (Baltim). 97:e12253, 2018).

**Case presentation:**

A 56-year-old man presented to our hospital with upper gastrointestinal bleeding and new-onset diabetes mellitus. He had no other medical comorbidities, episodes of pancreatitis and symptoms secondary to pancreatic insufficiency. A computed tomography (CT) scan showed a 94 × 72 × 83 mm necrotic pancreatic body mass with gastric invasion and multiple liver metastases. Gastroscopy revealed deep ulcerations at the posterior wall of the stomach with an active slow ooze. Endoscopic ultrasound was performed with EUS guided biopsy, which confirmed poorly differentiated squamous carcinoma of the pancreas. The patient underwent palliative radiotherapy for recurrent upper gastrointestinal bleeding followed by palliative chemotherapy with gemcitabine and nab-paclitaxel. He was referred to dietitians and diabetes educators for the management of pancreatic exocrine and endocrine insufficiency before being referred to community palliative care upon discharge.

**Conclusions:**

This is the first reported Australian case of pancreatic SCC presenting with upper gastrointestinal bleeding and new-onset diabetes mellitus. Patients with unresectable disease require a multidisciplinary approach to manage complications and improve symptom control. However, there are no standard treatment guidelines and future research is needed in this regard.

## Background

Pancreatic cancer is the seventh leading cause of cancer death worldwide [2]. It is estimated that it will become the third most leading cause of cancer death in Australia and the European Union [3, 4]. Most pancreatic cancers are malignant neoplasms of ductal lineage, with pancreatic ductal adenocarcinoma accounting for almost 90% of pancreatic neoplasms [5]. Primary squamous cell carcinoma (SCC) of the pancreas is an extremely rare type of cancer [6]. A review of the US Surveillance, Epidemiology, and End Results (SEER) database found 217 cases of primary pancreatic SCC, accounting for 0.2% of all pancreatic cancers [7]. The pathogenesis of pancreatic SCC remains unknown, although squamous metaplasia has been observed in up to 17% of cases in an autopsy study [8]. Squamous cells do not normally exist in the pancreatic parenchyma, and it is important to exclude any possibility of metastatic origin [5]. There is no standard treatment or guidelines available for the management of pancreatic SCC. In this report, we describe a rare case of unresectable primary pancreatic SCC in which the patient presented with upper gastrointestinal bleeding and new-onset diabetes mellitus (DM) and its challenges in subsequent management.

## Case report

**Fig. 1:**
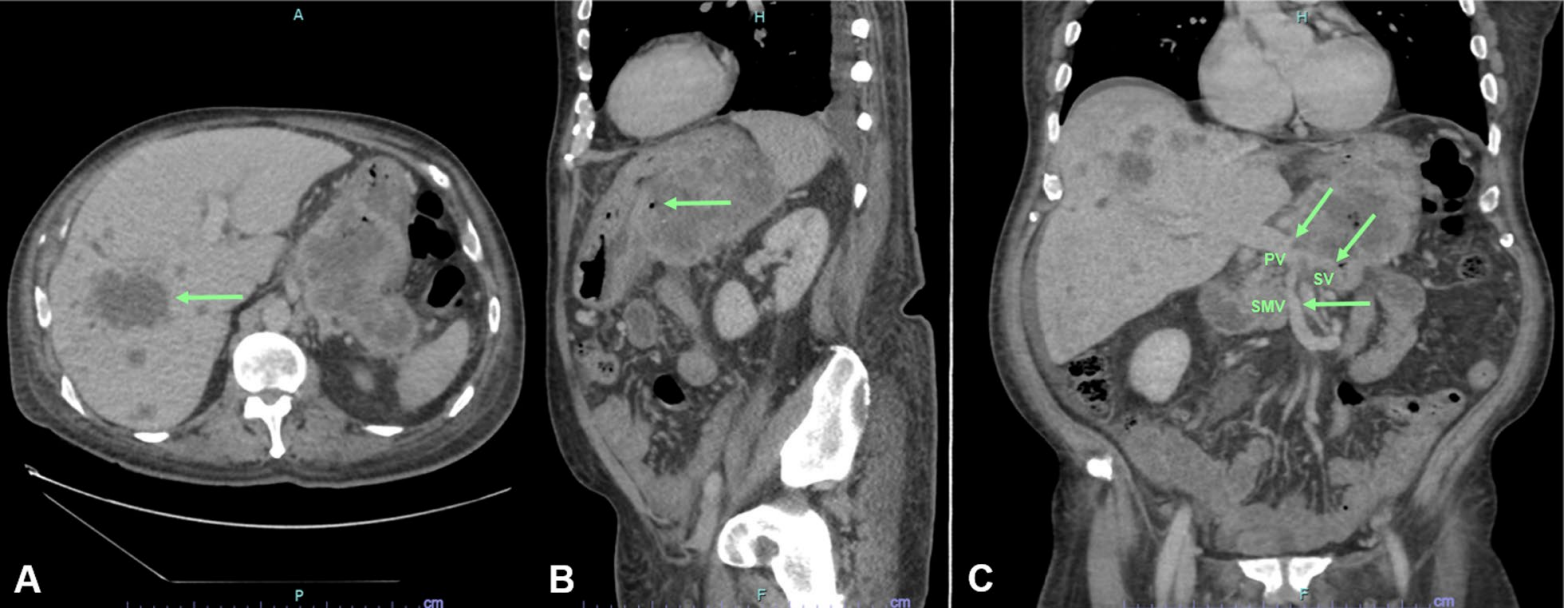
Abdominal pelvic CT scan showing a pancreatic body and tail SCC with multiple liver metastases. **A** Axial view demonstrating multiple hypoattenuating liver metastases with the largest lesion in segment 8 measuring 68 × 64 × 63 mm (arrow). **B** Sagittal view demonstrating the pancreatic mass compressing on the fundus and proximal gastric body with a small gaseous locule suggesting direct gastric invasion with possible fistulous formation (arrow), **C** coronal view showing the pancreatic SCC invading superior mesenteric vein (SMV), splenic vein (SV) and abutting the confluence of the portal vein (PV) by more than 180° (arrows)

**Fig. 2:**
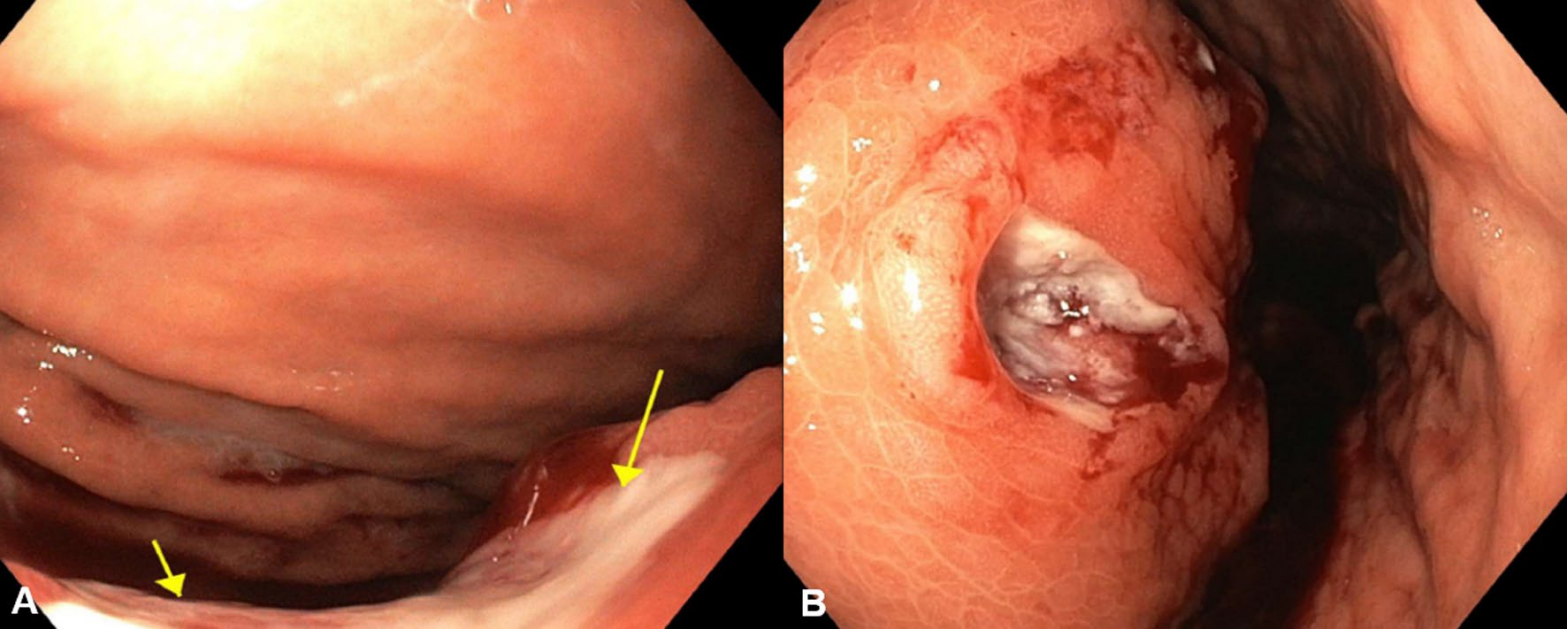
Gastroscopy showing (**A**) an ulcerated mucosa (yellow arrows) on a large inward indentation on the posterior wall of the stomach. **B** Retroflexion view demonstrating a second 25 mm deep ulceration on the lesser curvature of the stomach, which was friable with contact bleeding

**Fig. 3:**
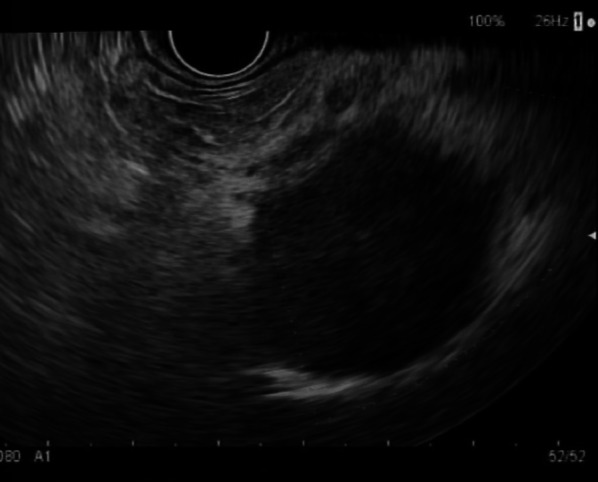
EUS demonstrating a large irregular hypoechoic paragastric lesion from the lesser curvature of the gastric body. EUS guided fine needle biopsy (FNB) was performed for histology

A 56-year-old male patient presented with hematemesis, melena, weight loss of 40 kg, steatorrhea, and new-onset DM. He had a history of left-sided abdominal pain for 10 years, which was managed conservatively. He had no other medical comorbidities; however, he had a family history of intra-abdominal malignancies of unknown primary origin in two first-degree relatives. His abdomen was tender in epigastrium and left upper quadrant with a palpable mass. There was no evidence of melena on digital rectal examination. His hemoglobin (Hb) was 100 g/L (Ref. 130–180) and his white cell count was elevated at 33.4 × 10^9^/L (Ref. 4.0–11.0) but urea, kidney, and liver function tests were normal. The heart rate and blood pressure were 110 beats per min and 103/63 mmHg, respectively, which responded to intravenous fluids. Computed tomography (CT) of the abdomen revealed a 94 × 72 × 83 mm necrotic pancreatic body mass with direct invasion of the superior mesenteric vein (SMV) and abutment of the portal vein (PV) by more than 180°. Gastric mural invasion at the lesser curvature and multiple liver metastases were also seen (Fig. 1). Staging CT unveiled no distant metastases. However, gastroscopy revealed two large deep ulcerations at the posterior wall of the stomach, which were friable and had slow ooze but no active bleeding. Given the nature of ulcers, no hemostatic procedure was performed; instead, a biopsy was taken to confirm the diagnosis (Fig. 2). Endoscopic ultrasound (EUS) revealed a large hypoechoic paragastric lesion at the lesser curvature of the gastric body (Fig. 3), and a fine-needle biopsy was performed. His Hb level steadily dropped to 84 g/L with ongoing small volume melena over the following week, requiring multiple transfusions with packed red blood cells.

**Fig. 4:**
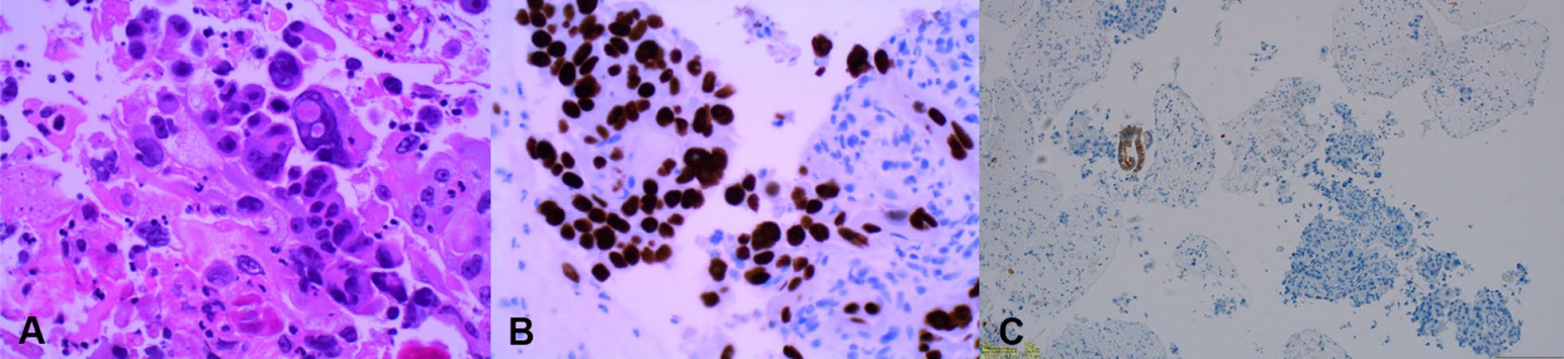
Histochemical photos of paragastric lesion biopsy from EUS showing features consistent with poorly differentiated pancreatic SCC. **A** Hematoxylin and Eosin stain demonstrating tumor cells with enlarged nuclei with moderate eosinophilic cytoplasm (magnification, × 40). **B** p40 immunohistochemistry highlighting a strong uptake by the tumor cells (magnification, × 40). **C** CK7 immunohistochemistry highlighting a single stripe of benign glandular tissue

Histological examination confirmed the diagnosis of poorly differentiated SCC of the pancreas with no adenocarcinoma components. The tumor cells were positive for p40 and CK 5/6 and negative for CK 20, CK 7, and CDX-2 (Fig. 4).

The objective of administering palliative radiotherapy was to control bleeding from the tumor, and a dose of 20 Gy in five fractions was delivered. The treatment was effective, as the patient did not experience any more episodes of melena, and his hemoglobin level remained stable at 90–100 g/L post-treatment.

Dietitians recommended dietary modifications for pancreatic exocrine insufficiency on cancer diagnosis. He was referred to diabetes educators and required a switch from oral hypoglycemic agents to insulin therapy because of worsening DM. The patient was linked to hospital and community palliative care providers during his inpatient stay.

The patient was subsequently considered for palliative chemotherapy as per a multidisciplinary recommendation. The Eastern Cooperative Oncology Group (ECOG) performance status was zero. The treatment protocol was initiated with a combination of gemcitabine and nab-paclitaxel every 4 weeks. Four days after the first cycle, he was admitted with non-neutropenic sepsis of unknown origin, with a fever of 41°, and hemodynamic instability requiring a brief period of vasopressor support. The Hb level was stable at 89 g/L, but WCC and neutrophil were elevated at 22.2 × 10^9^/L and 21.8 × 10^9^/L (Ref. 2.0–8.0), respectively. With no source of sepsis identified, he was discharged home on day four of hospital admission following supportive care. The patient’s second cycle of chemotherapy was cancelled after 4 weeks upon his request. Restaging CT showed disease progression. There was no evidence of pancreatic SCC regression, measuring 86 × 95 × 61 mm in size, but growth in numbers and sizes of liver metastases, measuring up to 68 mm and 58 mm in size, up from 17 and 12 mm, respectively. Four weeks later, the overdue chemotherapy was not administered as he presented with symptomatic anemia without evidence of melena. His ECOG performance status was four. Blood tests showed sepsis with an elevated WCC of 87 × 10^9^/L. No tumor markers were repeated. He was considered for comfort care by the palliative care team due to the high tumor burden, and he declined further chemotherapy. The patient succumbed to cancer 14 week post-diagnosis with sepsis.

## Discussion

Pancreatic SCC is an extremely rare pancreatic cancer, with an incidence of 0.5% [6, 9]. It is a unique cancer as the pancreas is devoid of squamous cells, and the histogenesis still remains unknown [1, 6, 9]. Several hypotheses have been proposed to explain the origin of cancer, including the malignant transformation of squamous metaplasia and squamous change in adenocarcinoma [1, 9]. It carries a poor prognosis with a median survival of 10 months and 4 months in resectable and unresectable cases, respectively. The symptoms include abdominal pain (78%), weight loss (57%), and jaundice (28%) [5]. Given its rarity, a thorough workup is required to exclude other possible primary metastatic SCC [1, 6, 9]. EUS-guided fine needle aspiration or biopsy remains the gold standard for the diagnosis of pancreatic SCC, since it was first reported by Lai et al. [1, 10] CT features of tumor enhancement with contrast and tumor blush patterns on angiography may help differentiate pancreatic SCC from adenocarcinoma [9]. Positron emission tomography–CT scans have been reported to be useful in excluding other metastatic sites. CA19-9 was elevated at 159 in our case, although it is a tumor marker that is often elevated in pancreatic adenocarcinoma [6].

There are no data supporting recommended chemotherapy regimens for pancreatic SCC and hence it is treated the same as adenocarcinoma by the NCCN guideline [11]. FOLFIRINOX (folinic acid, fluorouracil, irinotecan, and oxaliplatin) and gemcitabine with nab-paclitaxel are the two common first-line regimens for metastatic pancreatic adenocarcinoma, but the superiority of one over another has not been investigated with randomized trials. It makes it difficult for clinicians to choose which regimen should be the first line treatment for this subgroup of patients. At our institution, FOLFIRINOX is often offered to patients with resectable or borderline resectable pancreatic cancer, whereas gemcitabine-based therapy is often offered to patients with metastatic pancreatic cancer. Recently, the first cross-institutional retrospective study across 388 centers in the USA (*n* = 1102) showed that FOLFIRINOX was associated with more than 2 months of survival advantage over gemcitabine with nab-paclitaxel in this subgroup of pancreatic cancer [12]. A recent meta-analysis showed no significant difference between FOLFIRINOX and gemcitabine with nab-paclitaxel in terms of overall survival and progression-free survival. Subgroup analyses showed that the Asian subgroup had a better survival outcome with gemcitabine and nab-paclitaxel over FOLFIRINOX, whereas the Western subgroup had better survival with FOLFIRINOX [13]. The patient’s ethnicity may have been considered when gemcitabine-based therapy was chosen as the initial therapy in our case.

Only two cases have reported upper gastrointestinal bleeding caused by pancreatic SCC, and the bleeding was controlled by therapeutic endoscopy or resection [14, 15]. In our case, a short course of palliative radiotherapy of 20 Gy in five fractions was offered to control the patient’s abdominal pain and recurrent upper gastrointestinal bleeding post endoscopy. There are several treatment modalities to control upper gastrointestinal bleeding, including surgical resection, endoscopic hemostasis, and radiotherapy. Surgical resection was not recommended by our institutional multidisciplinary team as the patient had metastatic disease. Endoscopic intervention has been shown to achieve hemostasis in up to 67% of patients with advanced tumors of the stomach and duodenum, but the re-bleeding rate was high at 80% [16]. Endoscopic hemostasis is more likely to be successful in patients without diffuse bleeding and less advanced cancers [17].

Radiotherapy has shown benefits of palliation of bleeding due to advanced upper gastrointestinal tumors, including pancreatic adenocarcinoma [18]. If patients are hemodynamically stable enough to be transported to the radiotherapy department, radiotherapy can be delivered in a small number of treatments. A retrospective study on 112 patients who received palliative radiotherapy showed a bleeding control rate of 89% in gastrointestinal tumors. The overall re-bleeding rate was 25%, and the median time of re-bleeding was 84 days. The incidence of re-bleeding was not reduced with regimens consisting of more than five fractions (*p* = 0.65) [19]. A meta-analysis of palliative radiotherapy for symptomatic locally advanced gastric cancer showed a pooled overall response rate of 74% for hemostasis [20].

Up to 85% of patients with pancreatic adenocarcinoma have been shown to have impaired fasting glucose or diabetes mellitus [21]. One recent study showed that DM could be present in 52% of patients with unresectable pancreatic adenocarcinoma, in which 31% of patients had new-onset, and 69% had long-standing DM [22]. There is also a lack of data regarding the DM setting for pancreatic SCC. A systematic review of 54 cases showed that only one patient had been recently diagnosed with DM, and six patients had long-standing non-insulin-dependent DM [5]. The pathophysiologic association between diabetes mellitus and pancreatic adenocarcinoma remains largely unexplained. The mechanism of new onset DM in pancreatic adenocarcinoma is thought to be caused by paraneoplastic phenomena rather than mechanical injury to the pancreas itself. Some of the mediators include: (1) hyperglycemia and obesity causing insulin resistance, hyperinsulinemia promoting cell proliferation; (2) upregulated adrenomedullin mediating insulin resistance in β-cells; (3) cytokines from activated pancreatic stellate cells causing islet fibrosis and destruction; (4) pro-inflammatory adipokines mediating enhanced insulin resistance; and (5) increased lipolysis from excessive fat accumulation in the metabolic syndrome and obesity leading to generation of non-esterified fatty acids, development of insulin resistance and β-cells dysfunction [21, 23]. There is an association between tumor size and the degree of hyperglycemia [24]. While in type 2 diabetes mellitus there is an increased α-cell to β-cell ratio from amyloid deposition and a decrease in β-cell mass, in pancreatic cancer, both α-cell and β-cell islet size and density were found to be reduced [23]. To our knowledge, there is no literature describing the pathophysiologic association between pancreatic SCC and diabetes mellitus, and further research will be required.

## Conclusion

In conclusion, this study presents the first case of pancreatic SCC in Australia, presenting with upper gastrointestinal bleeding and new-onset DM. Given its rare incidence and poor prognosis, no standard treatment guideline currently exists. Palliative radiotherapy plays an important role in controlling hematemesis. Hence, patients with unresectable pancreatic SCC require a multidisciplinary approach to improve symptom control and quality of life.

## Data Availability

Data sharing is not applicable to this article as no data sets were generated or analyzed during the current study.

## Abbreviations

CT: Computed tomography
DM: Diabetes mellitus
ECOG: Eastern Cooperative Oncology Group
EUS: Endoscopic ultrasound
FOLFIRINOX: Folinic acid, fluorouracil, irinotecan and oxaliplatin
Hb: Hemoglobin
SV: Splenic vein
SCC: Squamous cell carcinoma
SMV: Superior mesenteric vein
PV: Portal vein
WCC: White cell count
